# Multiple Respiratory Syncytial Virus (RSV) Strains Infecting HEp-2 and A549 Cells Reveal Cell Line-Dependent Differences in Resistance to RSV Infection

**DOI:** 10.1128/jvi.01904-21

**Published:** 2022-03-14

**Authors:** Anubama Rajan, Felipe-Andrés Piedra, Letisha Aideyan, Trevor McBride, Matthew Robertson, Hannah L. Johnson, Gina Marie Aloisio, David Henke, Cristian Coarfa, Fabio Stossi, Vipin Kumar Menon, Harshavardhan Doddapaneni, Donna Marie Muzny, Sara Joan Javornik Cregeen, Kristi Louise Hoffman, Joseph Petrosino, Richard A. Gibbs, Vasanthi Avadhanula, Pedro A. Piedra

**Affiliations:** a Department of Molecular Virology and Microbiology, Baylor College of Medicine, Houston, Texas, USA; b Molecular and Cell Biology-Mol. Regulation, Baylor College of Medicine, Houston, Texas, USA; c Dan L Duncan Comprehensive Cancer Center, Baylor College of Medicine, Houston, Texas, USA; d Integrated Microscopy Core at the Gulf Coast Consortium Center for Advanced Microscopy and Image Informatics, Baylor College of Medicine, Houston, Texas, USA; e Department of Molecular and Human Genetics, Baylor College of Medicine, Houston, Texas, USA; f Department of Pediatrics, Baylor College of Medicine, Houston, Texas, USA; University of North Carolina at Chapel Hill

**Keywords:** A549, HEp-2, RSV, cytokines, host gene expression, viral gene expression

## Abstract

Respiratory syncytial virus (RSV) is a leading cause of pediatric acute respiratory infection worldwide. There are currently no approved vaccines or antivirals to combat RSV disease. A few transformed cell lines and two historic strains have been extensively used to study RSV. Here, we reported a thorough molecular and cell biological characterization of HEp-2 and A549 cells infected with one of four strains of RSV representing both major subgroups as well as historic and more contemporary genotypes (RSV/A/Tracy [GA1], RSV/A/Ontario [ON], RSV/B/18537 [GB1], and RSV/B/Buenos Aires [BA]) via measurements of viral replication kinetics and viral gene expression, immunofluorescence-based imaging of gross cellular morphology and cell-associated RSV, and measurements of host response, including transcriptional changes and levels of secreted cytokines and growth factors.

**IMPORTANCE** Infection with the respiratory syncytial virus (RSV) early in life is essentially guaranteed and can lead to severe disease. Most RSV studies have involved either of two historic RSV/A strains infecting one of two cell lines, HEp-2 or A549 cells. However, RSV contains ample variation within two evolving subgroups (A and B), and HEp-2 and A549 cell lines are genetically distinct. Here, we measured viral action and host response in both HEp-2 and A549 cells infected with four RSV strains from both subgroups and representing both historic and more contemporary strains. We discovered a subgroup-dependent difference in viral gene expression and found A549 cells were more potently antiviral and more sensitive, albeit subtly, to viral variation. Our findings revealed important differences between RSV subgroups and two widely used cell lines and provided baseline data for experiments with model systems better representative of natural RSV infection.

## INTRODUCTION

RSV contains substantial genetic diversity with two major subgroups, A and B, each containing multiple genotypes. Subgroups A and B are estimated to have diverged over 300 years ago (1) and show apparent convergent evolution over the last 10 to 20 years with the emergence of dominant genotypes containing nonoverlapping but adjacent duplications of less than 100 nucleotides (72 nucleotides in RSV/A; 60 nucleotides in RSV/B) within the second hypervariable region of the G gene (2). Although this duplication appears to result in a modest enhancement of RSV binding to host cells (3), its full effects on the viral life cycle and host response are unknown. Furthermore, whole genomes of historic RSV strains (i.e., those isolated over 20 years ago) and more contemporary strains from the same subgroup show a sequence divergence of ∼5%, which is ∼4-fold lower than that of cognate strains (historic or contemporary) from different subgroups. Thus, genetic variation in RSV is substantial in magnitude and interesting in structure. It remains unknown whether the genetic variation separating RSV subgroups and genotypes leads to significant functional differences in the RSV life cycle and whether or to what extent it provokes divergent host responses.

Immortalized respiratory epithelial cell lines, particularly HEp-2 and A549, have been used to study RSV for decades (4–10). HEp-2 cells were derived from a larynx carcinoma over 60 years ago, but the publicly available current cell line shows evidence of contamination with HeLa cells (11–14). A549 cells were derived from a type II alveolar epithelial carcinoma and show no signs of contamination with another cell line. To date, no study has systematically searched for differences between these widely used and seemingly interchangeable cell lines with respect to their permissiveness to RSV infection, their RSV-induced cellular and immune responses, and their sensitivity to different RSV strains.

Here, we reported a broad and thorough characterization of *in vitro* RSV infections using a diverse set of viral strains in two major cell lines. We infected HEp-2 and A549 cells with four RSV strains belonging to both major subgroups and four genotypes representing both historic (RSV/A/Tracy [GA1 genotype], and RSV/B/18537 [GB1 genotype]) and contemporary strains (RSV/A/Ontario [ON genotype] and RSV/B/Buenos Aires [BA genotype]). We assessed (i) viral replication kinetics and viral gene expression, (ii) the distribution of infecting and/or egressing RSV and accompanying cell morphological changes, (iii) host transcriptional changes, and (iv) levels of secreted cytokines and growth factors. Our data indicated that HEp-2 and A549 cells differed systematically in their response to RSV infection with the latter cell line supporting less viral replication by mounting a more effective antiviral response. Interestingly, facets of the A549 response appeared to support the RSV life cycle in ways the HEp-2 response did not, and A549 cells showed heightened sensitivity to differences within infecting RSV strains.

## RESULTS

### HEp-2 cells support greater RSV replication than A549 cells.

**FIG 1:**
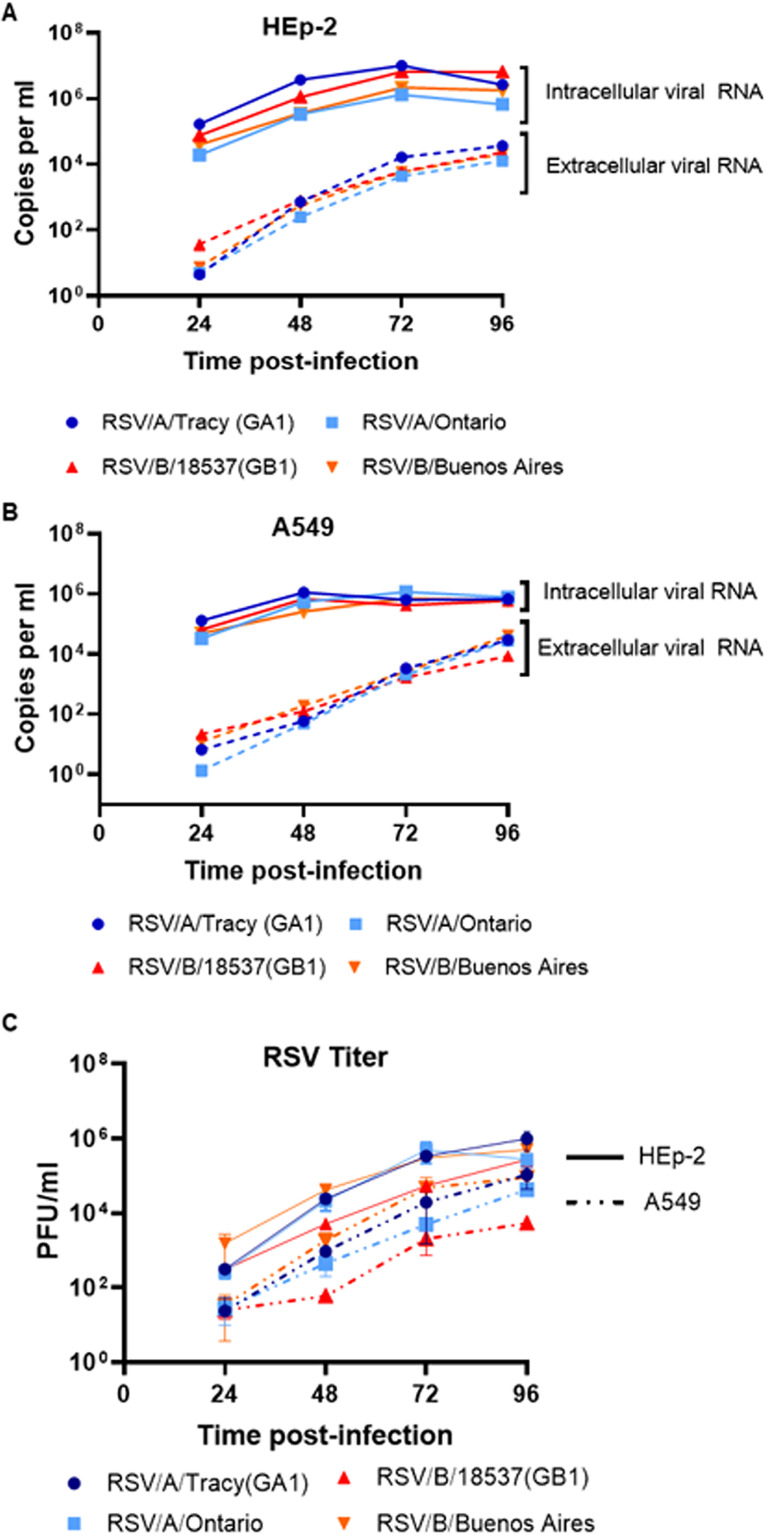
Viral load and replication kinetics of RSV infection. HEp-2 and A549 cells were infected with RSV (RSV/A/Tracy [GA1], RSV/A/Ontario [ON], RSV/B/18537 [GB1], RSVB/Buenos Aires [BA]) at a multiplicity of infection (MOI) of 0.01. Samples were collected at 24, 48, 72, and 96 h postinoculation (hpi). RNA was isolated from media (extracellular) and cell lysate (intracellular) and copy numbers of RSV nucleocapsid (N) gene RNA were determined using quantitative real-time PCR (qRT-PCR). Levels of RSV N gene RNA in (A) HEp-2 cells at different time points after RSV inoculation, and (B) A549 cells at different time points after RSV inoculation. (C) Extracellular live virus was collected from the media of HEp-2 and A549 inoculated cell cultures. The extracellular virus concentrations were determined by a quantitative plaque assay and reported as log_10_ plaque forming unit (PFU)/mL in HEp-2 cells. Data shown are from two individual experiments with two replicates per group in each experiment and are represented as mean ± SD.

HEp-2 and A549 cells were compared for their ability to support replication of the four RSV strains (RSV/A/Tracy [GA1 genotype], RSV/A/Ontario [ON genotype], RSV/B/18537 [GB1 genotype], RSV/B/Buenos Aires [BA genotype]) (Fig. 1). HEp-2 cells showed significantly greater mean levels of intracellular and extracellular viral RNA through time and across strains (Fig. 1A and B) (linear regression model, *P* < 0.01). Consistent with viral RNA levels, the number of infectious virions released into the extracellular fluid was greater from HEp-2 cells than A549 cells through time and across strains (Fig. 1C) (linear regression model, *P* < 0.01). There were no significant differences in overall growth kinetics (levels of viral RNA or infectious virions) between RSV subgroups or historic (RSV/A/Tracy [GA1] and RSV/B/18537 [GB1]) and contemporary (RSV/A/Ontario [ON] and RSV/B/Buenos Aires [BA]) strains in A549 cells. However, historic strains showed higher levels of intracellular viral RNA than contemporary strains in HEp-2 cells (two-way ANOVA with Tukey’s multiple-comparison test, *P* = 0.01).

### Viral gene expression was comparable across strains, but transcriptional readthrough showed a major difference between RSV subgroups A and B.

**FIG 2:**
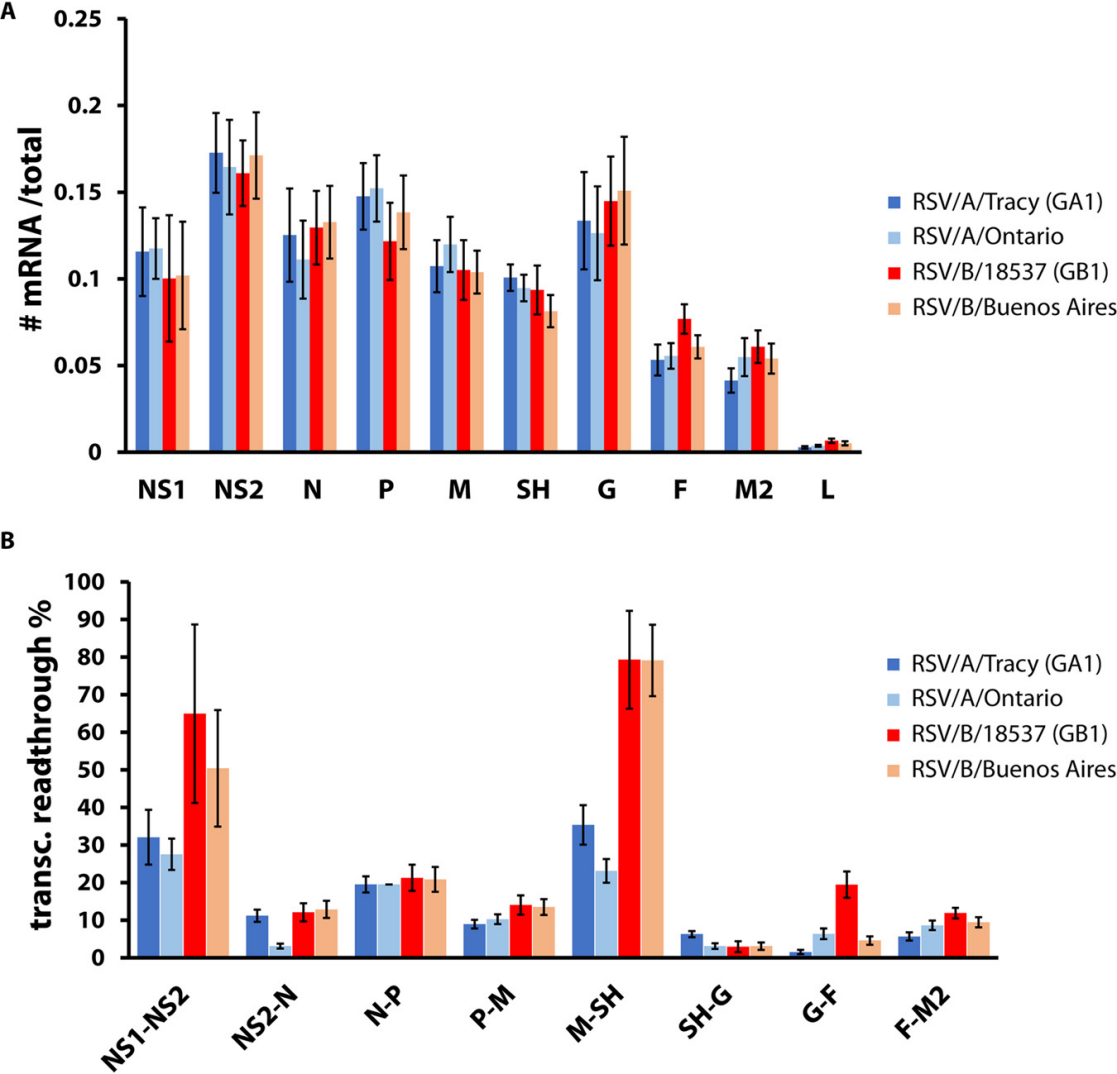
RSV gene expression. HEp-2 cells were infected with RSV (RSV/A/Tracy [GA1], RSV/A/Ontario, RSV/B/18537 [GB1], RSV/B/Buenos Aires) at a multiplicity of infection (MOI) of 0.01. Samples were collected at 24 and 48 h postinoculation (hpi). RNA was isolated from cell lysates and prepared for and subjected to high-throughput short-read sequencing. (A) Relative mRNA levels are comparable across the four RSV strains tested. For each time point and each of 10 RSV genes, the average read depth across the gene’s coding sequence was divided by the total number of reads mapping to all 10 viral coding sequences. Data shown are averages ± SD of results for 24 and 48 hpi. (B) Transcriptional readthrough at particular gene junctions varied between RSV subgroups and strains. For each time point and each of eight RSV gene junctions, the average read depth across the gene junction was divided by the average read depth across the nearest upstream coding sequence. Data shown are averages ± SD of results for 24 and 48 hpi.

We measured genome-wide transcription from RSV strains using high-throughput short-read mRNA sequencing (mRNAseq). Previous measurements by qPCR showed that viral gene expression patterns do not vary by cell line (15), so we employed the more widely used HEp-2 cells instead of A549 cells for the measurements reported here. RSV-infected cell lysate samples were collected for mRNAseq at time points (24 and 48 h postinoculation [hpi]) occurring within a previously established window of steady-state gene expression (15). The resulting coverage plots were averaged to reveal a genome-wide transcription pattern for each infecting strain. Genome-wide transcription patterns were comparable across strains, showing what appeared to be three tiers of gene expression (high: NS1-G; medium: F-M2; low: L) (Fig. 2A). Percentages of transcriptional readthrough at each gene junction were mostly comparable among strains but showed large differences at NS2-N, M-SH, and G-F gene junctions (Fig. 2B). The M-SH gene junction showed a large subgroup-dependent difference (Fig. 2B). An apparent subgroup-dependent difference in readthrough also occurred at the NS1-NS2 gene junction, but the higher percentage and large error recorded for both subgroup B viruses result from a dip in sequence coverage over the NS1 open reading frame (ORF) (unpublished data) of unknown origin (Fig. 2B). For all strains, the NS1-NS2, N-P, and M-SH gene junctions had the highest percentages of transcriptional readthrough.

### Distribution of RSV and gross morphological changes via rearrangements of the actin cytoskeleton in infected HEp-2 and A549 cells.

**FIG 3:**
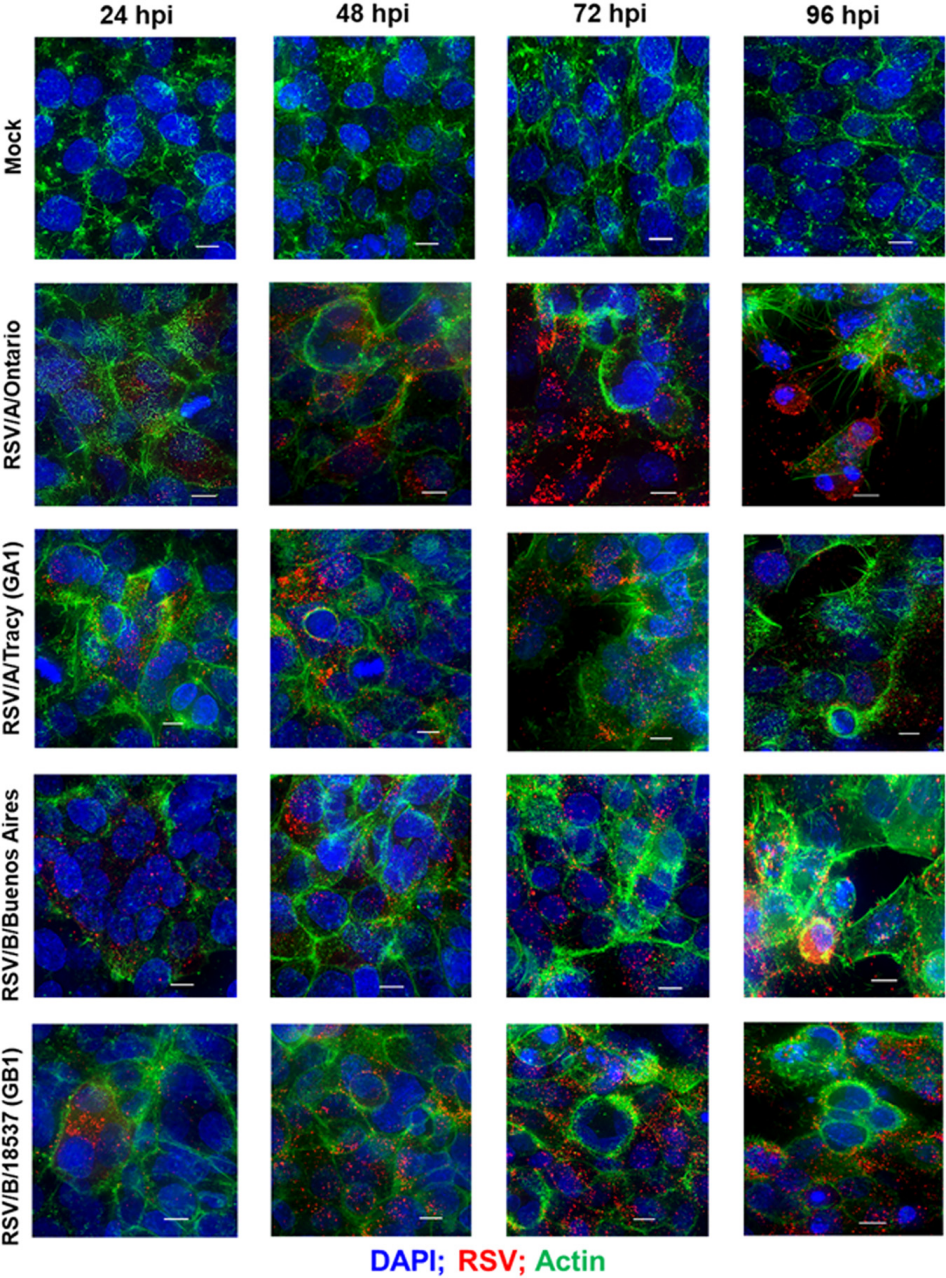
RSV infectivity pattern and cellular damage in HEp-2 cells visualized by immunofluorescence micrographs. Representative epifluorescence deconvolution micrographs of HEp-2 cells labeled or nuclei (DAPI), RSV (M2-1; red), and Actin (green). Cells were either mock-treated or infected with RSV/A/Tracy (GA1), RSV/A/Ontario (ON), RSV/B/18537 (GB1), or RSV/B/Buenos Aires (BA) at a multiplicity of infection of 0.01 for 24, 48, 72, or 96 h. Scale bars indicate 10 μm.

**FIG 4:**
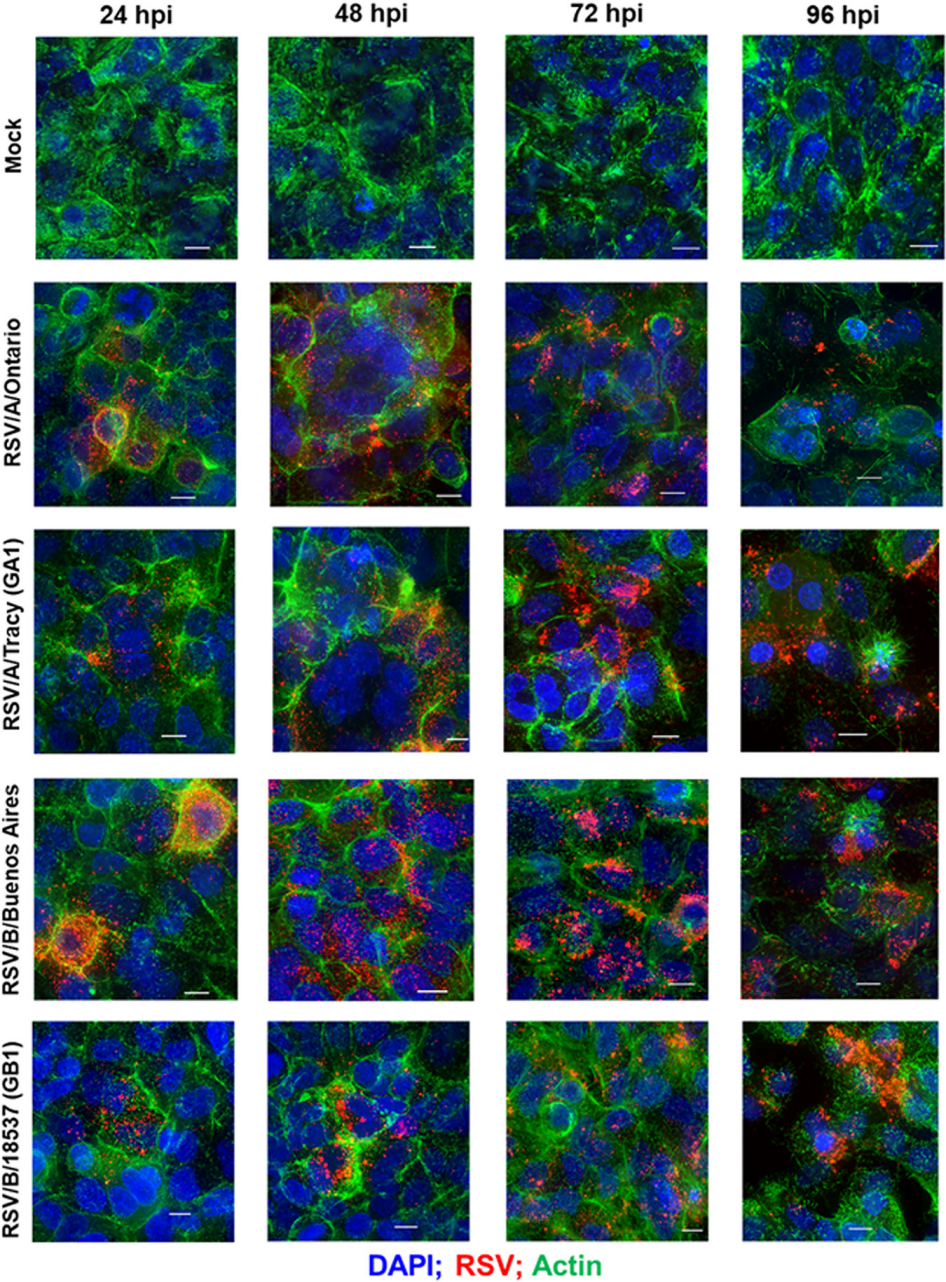
RSV infectivity pattern and cellular damage in A549 cells visualized by immunofluorescence micrographs. Representative epifluorescence deconvolution micrographs of A549 cells labeled for nuclei (DAPI), RSV (M2-1; red), and Actin (green). Cells were either mock-treated or infected with RSV/A/Tracy (GA1), RSV/A/Ontario (ON), RSV/B/18537 (GB1), or RSV/B/Buenos Aires (BA) at a multiplicity of infection of 0.01 for 24, 48, 72, or 96 h. Scale bars indicate 10 μm.

To explore how RSV infects and spreads and whether it induces changes to the cytoskeletal structure in a cell line- and/or RSV strain-dependent way, we infected HEp-2 and A549 cells with RSV strains at low multiplicity of infection (MOI; 0.01) and performed epifluorescence deconvolution imaging for actin, cell nuclei, and RSV at 24, 48, 72, and 96 hpi. Regardless of strain, HEp-2 cells showed widespread infection at 24 hpi with RSV levels increasing before reaching a plateau at 72 hpi (Fig. 3). In contrast, RSV infection in A549 cells was limited to small foci of cells at 24 hpi with subsequent spreading comparable to that seen in HEp-2 cells (Fig. 4).

**FIG 5:**
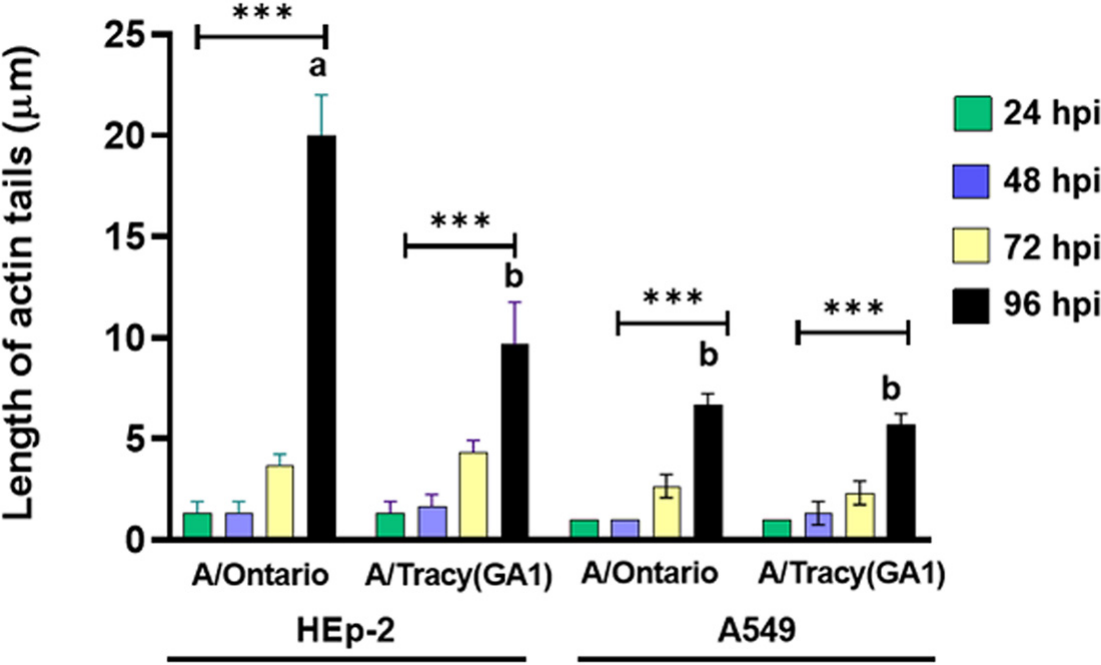
Quantification of the length of actin tails in RSV/A-infected HEp-2 and A549 cells. Data shown were gathered from three independent samples with three replicates per group in each experiment and are represented as mean ± SD. Asterisks indicate statistical significance determined by two-way ANOVA with Tukey’s multiple-comparison test, *P* < 0.001 (***). Bars with different letters are significantly different from each other, *P* < 0.01.

Actin staining revealed differences in actin rearrangement between infected HEp-2 and A549 cells and differing rearrangements induced by RSV subgroups A and B (Fig. 3 and 4). Mock-infected HEp-2 and A549 cells also differed in the morphology of their actin cytoskeleton with A549 cells containing a larger number of actin filaments or filament bundles of greater size. Both cell lines showed actin rearrangements in response to all four infecting strains at the earliest time point visualized, 24 hpi, with actin filaments adopting an arrangement reminiscent of thin halos at the boundaries of infected cells. In HEp-2 cells infected with RSV/B strains, the actin halos increased in thickness at 72 hpi and beyond. In HEp-2 cells infected with RSV/A strains, actin halos rearranged into microns-long tail-like structures at 96 hpi (Fig. 3 and 5). In addition, RSV/A-infected HEp-2 cells showed considerable cytopathic effect at 96 hpi while RSV/B-infected HEp-2 cells did not. In contrast, RSV-infected A549 cells displayed actin rearrangements resulting in what appear to be paracellular gaps (i.e., loss of cellular tight junctions due to actin rearrangements) rather than cellular damage at 96 hpi (Fig. 4).

### Global changes in HEp-2 and A549 gene expression in response to RSV infection.

**FIG 6:**
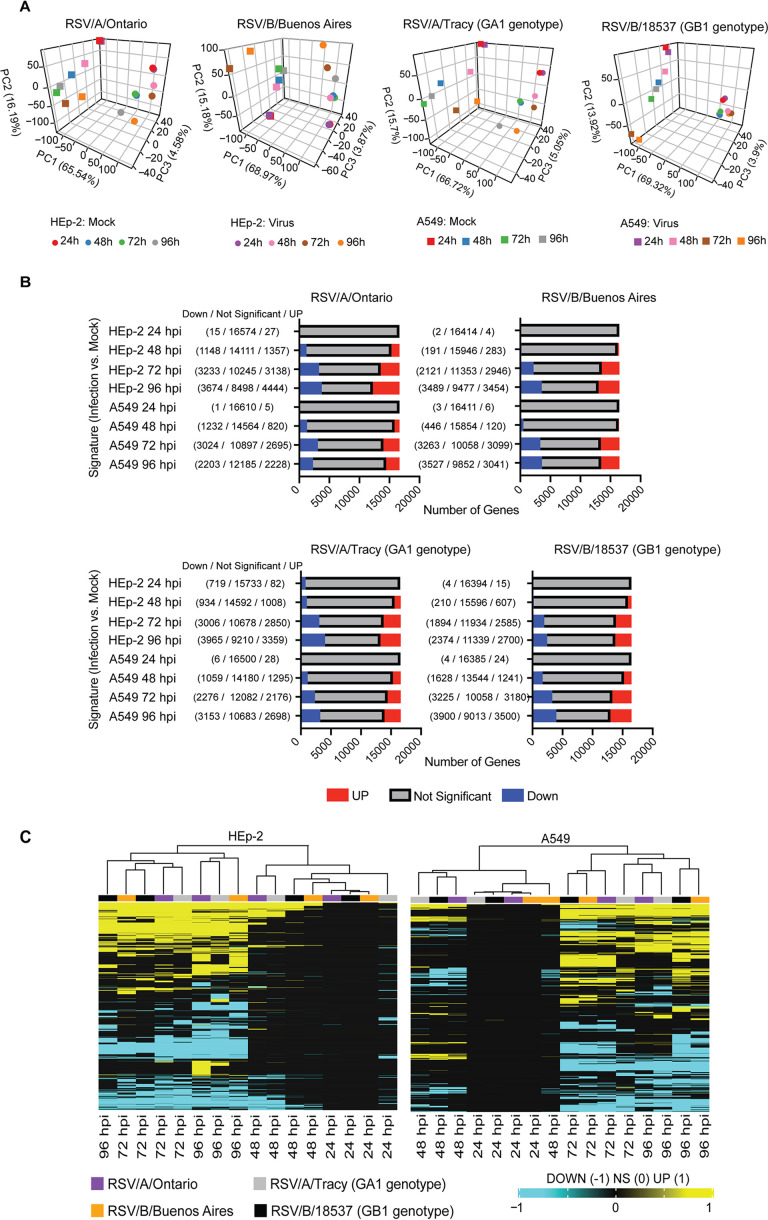
RNA sequencing analysis of RSV infections of HEp-2 and A549 cells. (A) Principal-component analysis of RSV/A/Ontario- (ON), RSV/B/Buenos Aires- (BA), RSV/A/Tracy- (GA1), and RSV/B/18537- (GB1) infected HEp-2 and A549 cells demonstrating the variability observed in the samples (HEp-2 versus A549 and infected versus mock-infected controls). (B) Total number of significant genes identified for the RSV/A/Ontario-, RSV/B/Buenos Aires-, RSV/A/Tracy-, and RSV/B/18537-infected group in HEp-2 and A549 cells (adjusted *P* < 0.05). (C) Samples were hierarchically clustered based on the union of differentially expressed genes across all comparisons (false discovery rate [FDR] <0.05 and fold change exceeding 1.5×). Genes were converted to 1 for upregulated; −1 for downregulated; and 0 for no significant upregulation or downregulation in a particular signature. Samples were clustered using the Euclidean distance.

We sought to determine whether any of the differences observed in RSV infection could be explained by the host transcriptional response. To do so, we subjected RSV-infected and mock-infected A549 and HEp2 cells to high-throughput, short-read RNA sequencing (RNAseq). Principal-component analysis (PCA) showed that cell type accounted for most of the variation in the data (Fig. 6A), and a strong transcriptional response to RSV in both HEp-2 and A549 cells did not become clear until 72 and 96 hpi regardless of infecting strain (Fig. 6A). Of the 4 time points investigated, the number of significantly upregulated and downregulated genes relative to mock-infected cells was low at 24 hpi (<100) then rose rapidly beyond 48 hpi before approaching a peak of ∼6000 to 8000 genes at 72 hpi and beyond (Fig. 6B). Upregulated and downregulated genes were clustered into a heat map to compare across cell lines, virus strains, and time points, demonstrating similar host transcriptional kinetic profiles (Fig. 6C).

### Functional analysis revealed many cell line-dependent and some viral strain-dependent features of the host transcriptional response to RSV infection.

**FIG 7:**
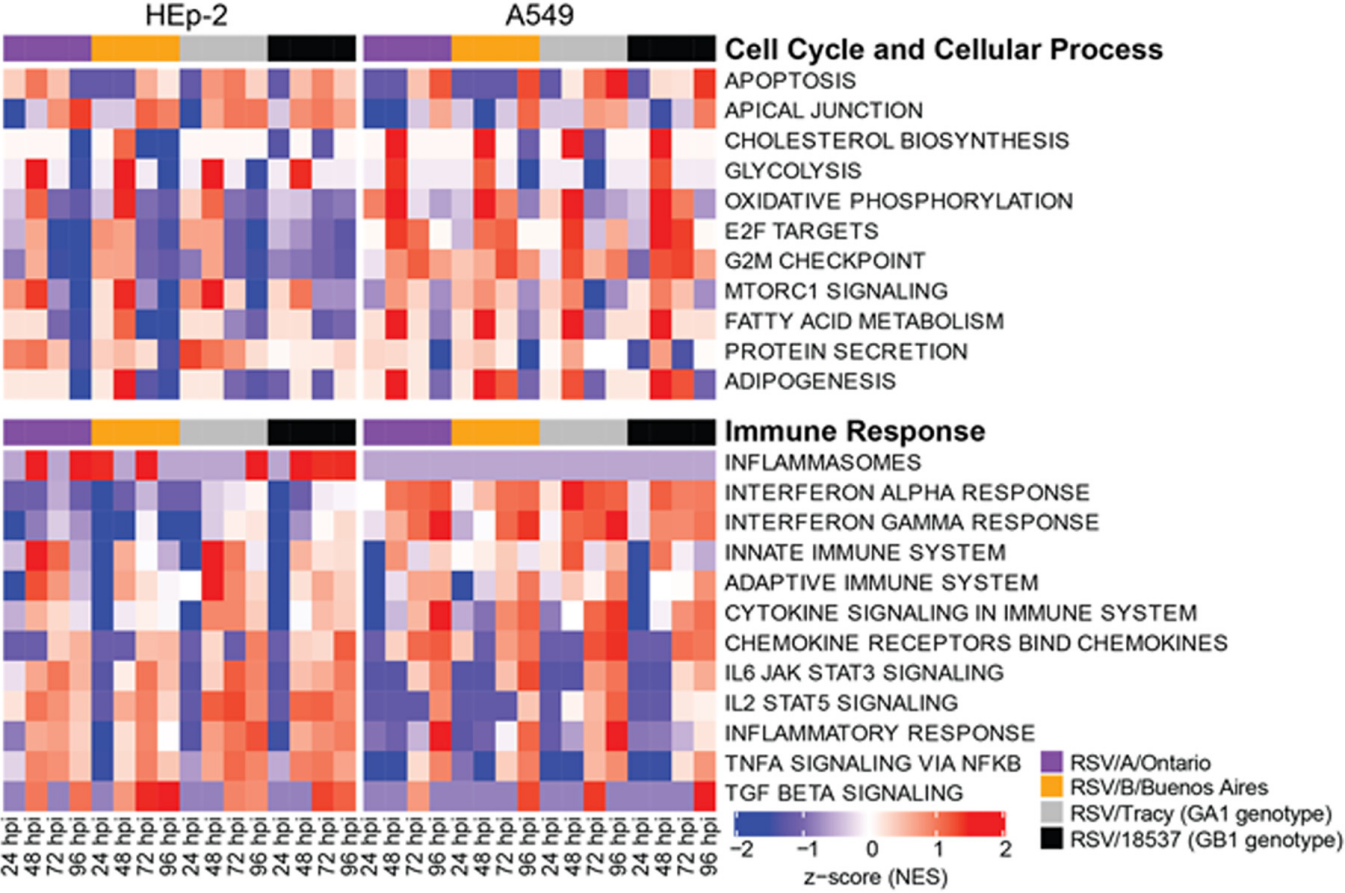
Select reactome and hallmark gene ontology pathways identified from a gene set enrichment analysis by filtering for the top 10 normalized enrichment scores (NES) for each category. A false discovery rate of 0.05 was used in the pathway filtering.

**FIG 8:**
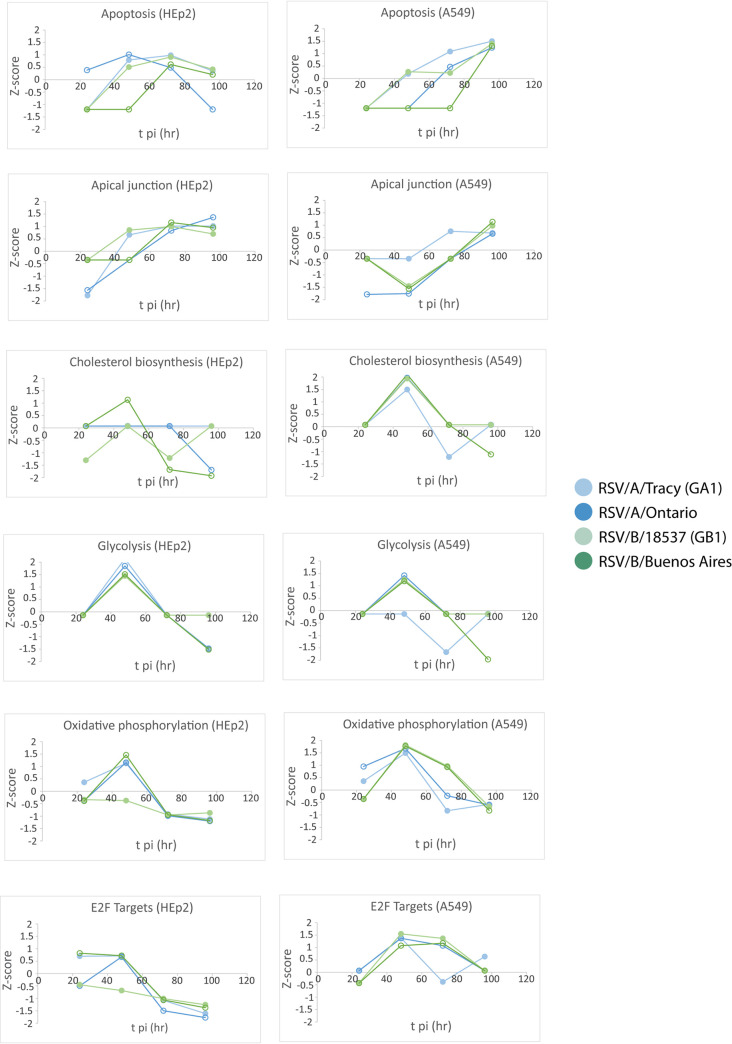
Normalized enrichment scores (NES) through time for gene set enrichment analysis (GSEA) identified cellular and metabolic pathways enriched for upregulated or downregulated genes relative to mock.

**FIG 9:**
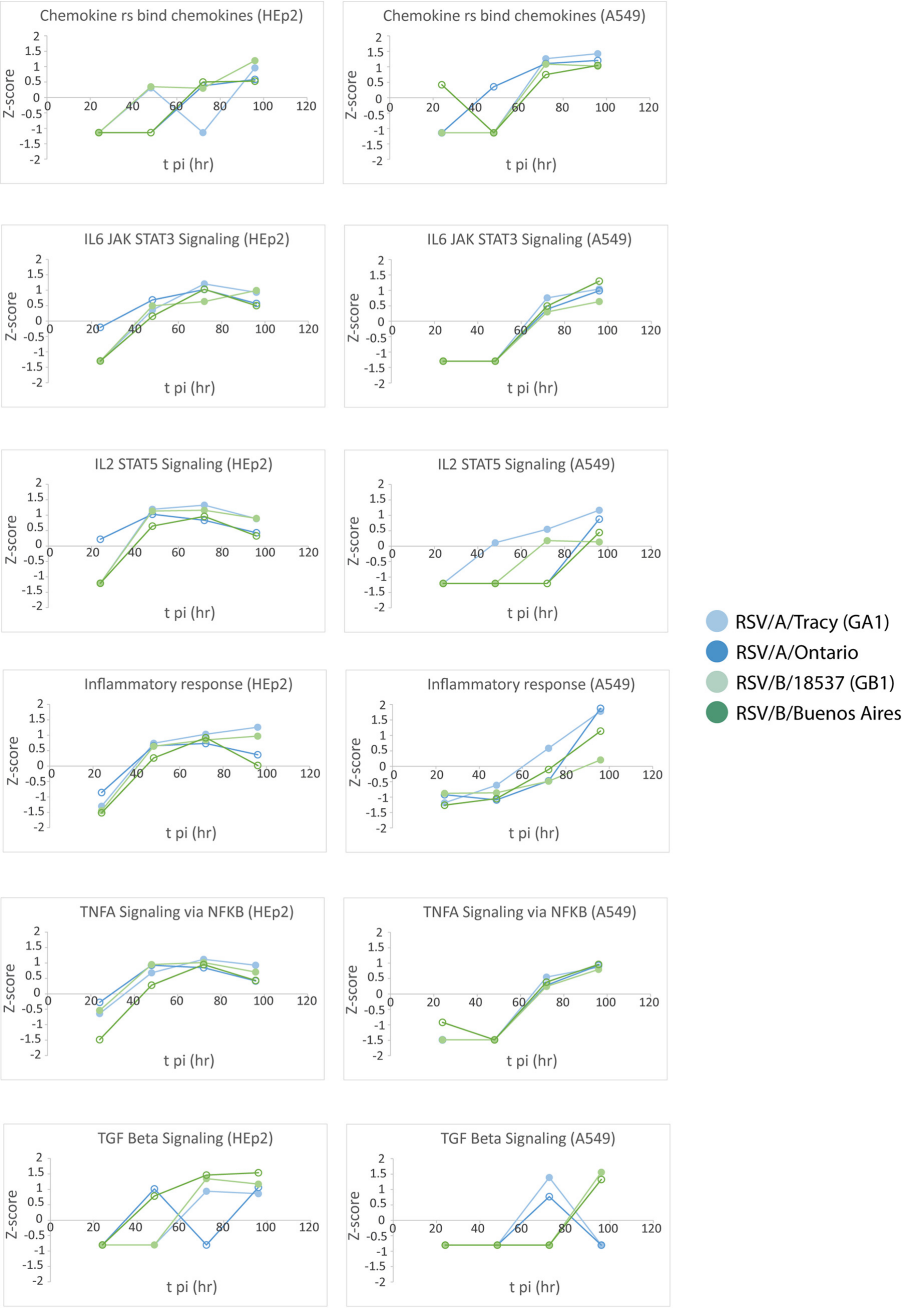
Normalized enrichment scores (NES) through time for gene set enrichment analysis (GSEA) identified immune pathways enriched for upregulated or downregulated genes relative to mock.

**FIG 10:**
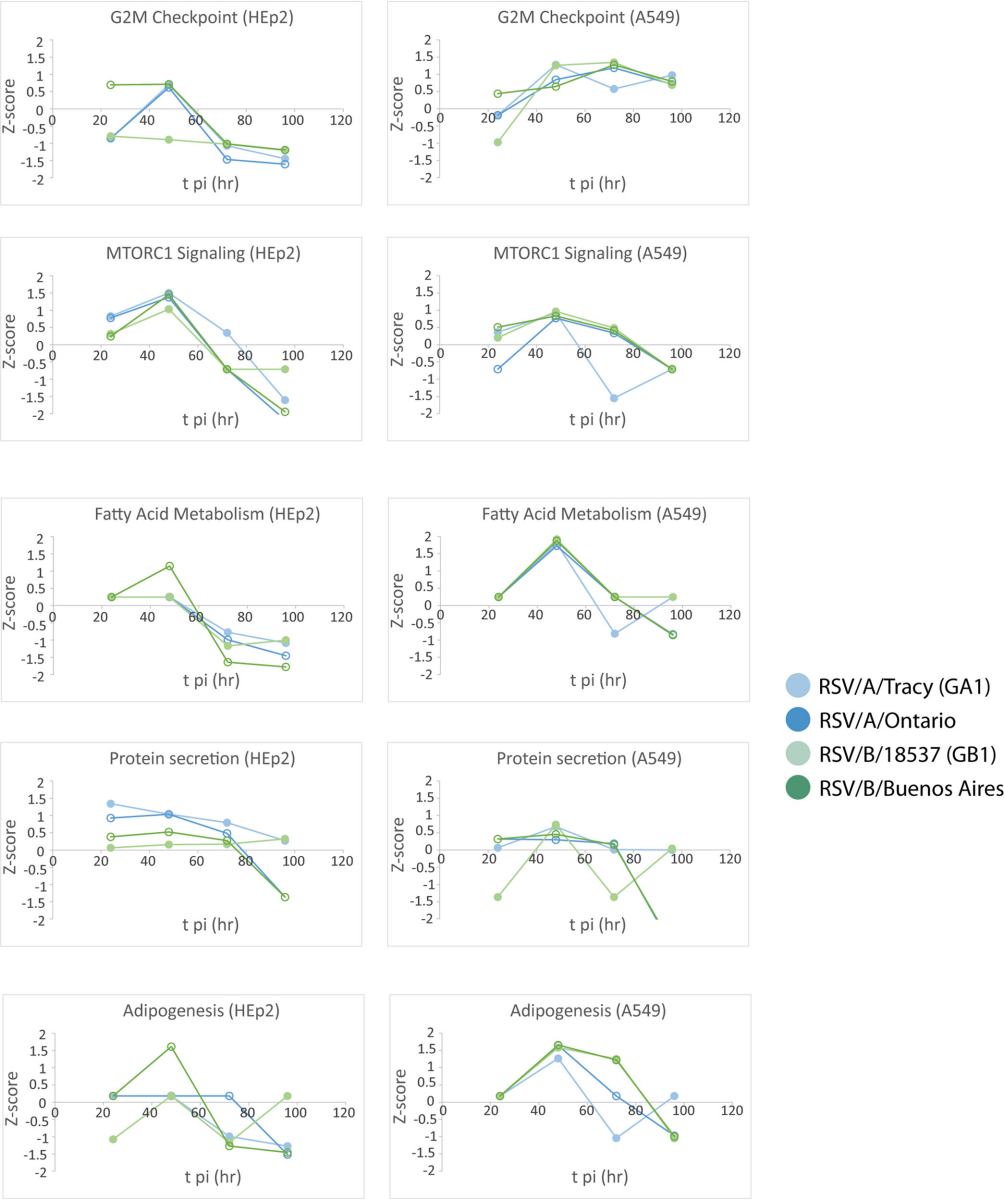
Normalized enrichment scores (NES) through time for gene set enrichment analysis (GSEA) identified cellular and metabolic pathways enriched for upregulated or downregulated genes relative to mock.

**FIG 11:**
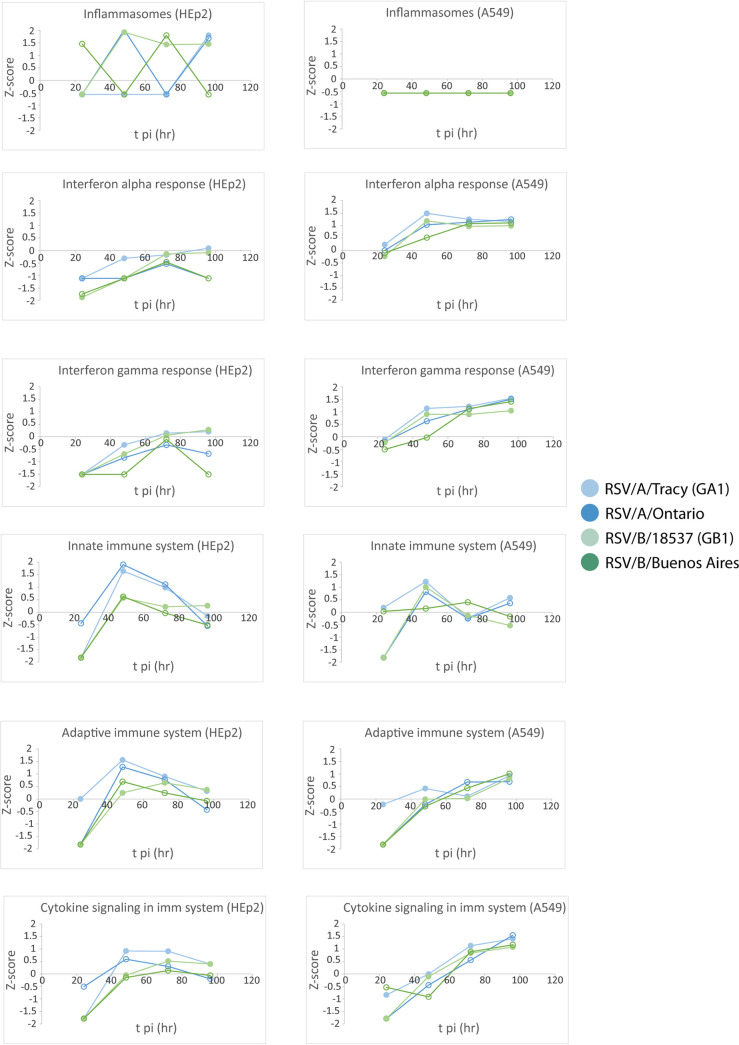
Normalized enrichment scores (NES) through time for gene set enrichment analysis (GSEA) identified immune pathways enriched for upregulated or downregulated genes relative to mock.

The gene set enrichment analysis (GSEA) software (16) was used to search our data for functional pathways enriched for upregulated or downregulated genes relative to mock-infected cells (≥2× upregulated or downregulated and false discovery rate [FDR] < 0.05). The pathways identified were mostly related to host immune response, cell cycle, and metabolic processes (Fig. 7 to 9). Many of the functional pathways identified show normalized enrichment scores (NES) with temporal dynamics that differed strongly between HEp-2 and A549 cells. For instance, the NES for the apoptotic response in A549 cells rose monotonically to a peak at 96 hpi regardless of the infecting strain, while the HEp-2 apoptotic response was weaker and dipped to near baseline values at 96 hpi (Fig. 7 and 8). Genes involved in progression through the cell cycle (E2F target and G2M checkpoint genes) were upregulated at 48 and 72 hpi in A549 cells, and they were strongly repressed at 72 and 96 hpi in HEp-2 cells (Fig. 10). Genes upregulated in response to interferon-alpha (IFN-α) and interferon-gamma (IFN-γ) were activated beyond 24 hpi in A549 cells, and they were strongly repressed at 24 hpi in HEp2 cells with the NES gradually rising to <0.5 at 96 hpi for all four RSV strains assayed (Fig. 7 and 11). Genes encoding components of the inflammasome appeared highly upregulated during at least one time point for HEp-2 cells infected with any of the four RSV strains, while their expression state did not differ significantly from mock in A549 cells (Fig. 11). The NES for genes involved in cytokine signaling rose monotonically to a peak of 1 to 1.5 at 96 hpi in A549 cells but plateaued at lower values between 48 and 72 hpi in HEp-2 cells, with RSV/A/Tracy-infected HEp-2 cells being the only combination showing modest upregulation (NES ∼0.9) at both 48 and 72 hpi in HEp-2 cells (Fig. 11). Genes upregulated in response to increases in IL-2 and IL-6 via STAT5 and STAT3, respectively, showed mostly switch-like, but modest, activation between 48 and 72 hpi in A549 cells while showing gradual and comparably modest activation plateauing beyond 72 hpi in HEp-2 cells (Fig. 7 and 9). The NES for genes involved in the inflammatory response gradually rose through the course of the experiment in A549 cells with the two RSV/A infections showing significant peak upregulation (NES 1.8 to 1.9) at 96 hpi. In contrast, the NES plateaued at more modest values beyond 48 hpi in infected HEp-2 cells (Fig. 9). Genes upregulated by NF-κB in response to TNF-α had an NES that increased in switch-like fashion beyond 48 hpi in A549 cells, rising to modest activation (NES 0.8 to 1) at 96 hpi. The NES for these genes was higher at and plateaus beyond 48 hpi in infected HEp-2 cells (Fig. 9).

The two cell lines also showed highly similar responses to RSV in a few pathways (Fig. 7 to 10). Both showed (i) gradual activation of genes encoding components of the apical junction complex; (ii) significant upregulation of glycolysis and oxidative phosphorylation at 48 hpi with two minor exceptions showing no upregulation at that time point (glycolysis: RSV/A/Tracy:A549; oxidative phosphorylation: RSV/B/18537:HEp-2); and (iii) gradually increasing activation of chemokine receptor genes through 96 hpi.

Some metabolic pathways even showed a host response in apparent favor of the viral life cycle: genes associated with cholesterol biosynthesis (Fig. 8), fatty acid metabolism (Fig. 10), and adipogenesis (Fig. 10) were highly upregulated regardless of infecting strain in A549 cells at 48 hpi. A similar but lower peak in upregulation occurred in HEp-2 cells but only in response to the RSV/B/Buenos Aires strain.

We also observed a few instances of apparent RSV subgroup-dependent and historical versus contemporary strain-dependent differences in host response, especially in A549 cells. In response to the two RSV/A strains assayed, genes upregulated in response to TGF-β1 were strongly activated at 72 hpi before reverting to a repressed state at 96 hpi. In response to the two RSV/B strains assayed, these genes remained repressed until 96 hpi when they switched to being strongly upregulated (Fig. 9). Both the expression status of genes upregulated by STAT5 in response to IL-2 stimulation (Fig. 9) and genes involved in protein secretion (Fig. 10) showed an apparent dependence in A549 cells on whether the infecting RSV strain was historic or contemporary. The latter also seemed to hold in HEp2 cells. In both cell lines, genes associated with protein secretion were strongly downregulated at 96 hpi only when the infecting strain was contemporary (RSV/A/Ontario or RSV/B/Buenos Aires).

### Cytokine profiles of HEp-2 and A549 cells in response to RSV infection.

**FIG 12:**
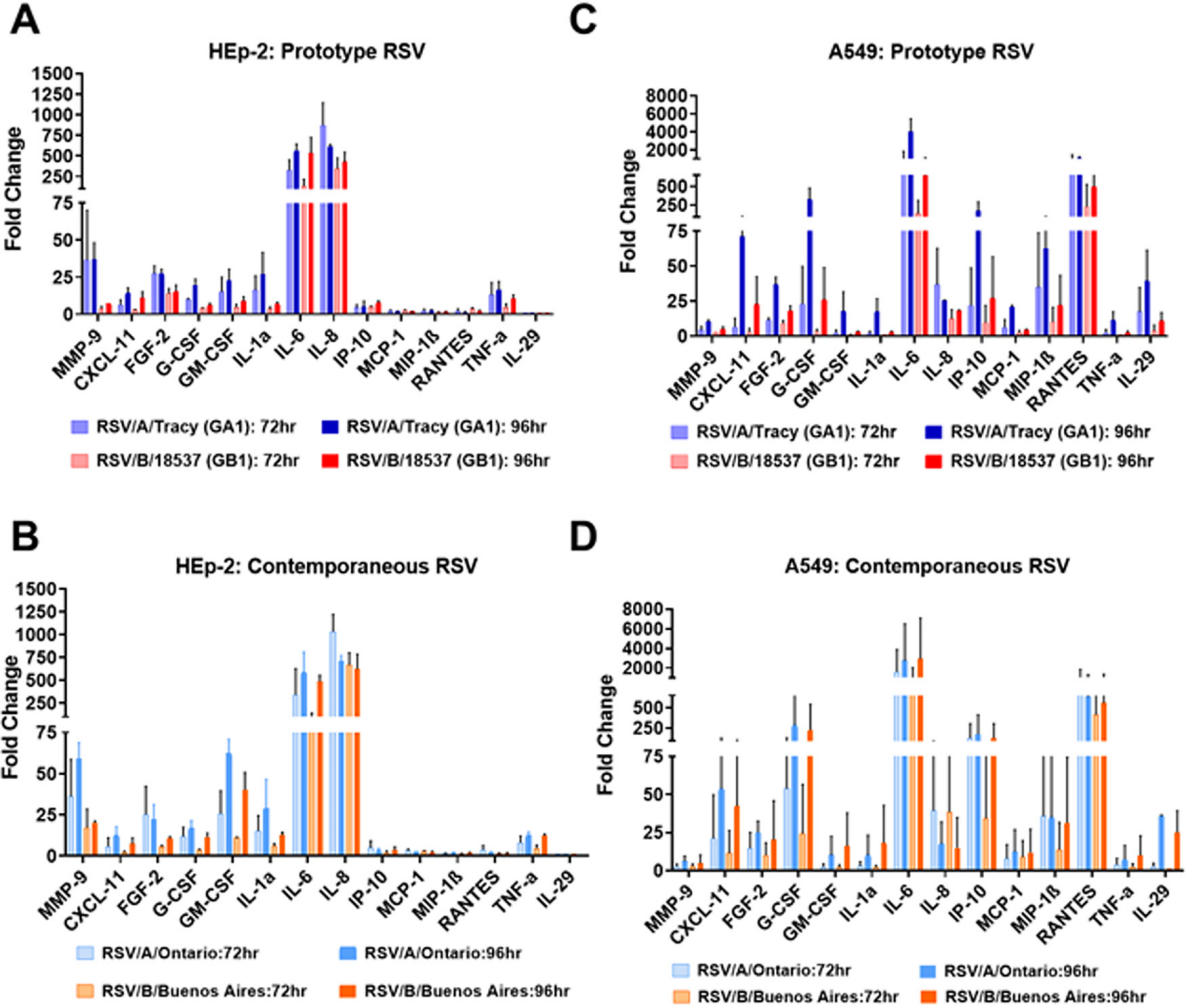
Profiles of released cytokines and chemokines from HEp-2 and A549 cells. Cells were infected with RSV/A/Tracy (GA1), RSV/B/18537 (GB1), RSV/A/Ontario (ON), or RSV/B/Buenos Aires (BA) at a multiplicity of infection (MOI) of 0.01. The cultured supernatants were harvested from mock-infected and RSV-infected cells at 24, 48, 72, or 96 h postinoculation (hpi). Profiles of cytokines and chemokines released into the supernatant were determined by a multiplex Luminex cytokine assay. The fold change of each cytokine or chemokine between virus- and mock-infected cells are shown. Fold changes of cytokines and chemokines released from HEp-2 cells infected with (A) historic RSV strains (RSV/A/Tracy (GA1) and RSV/B/18537 (GB1)) and (B) contemporary RSV strains (RSV/A/Ontario [ON] and RSV/B/Buenos Aires). Fold changes of cytokines and chemokines released from A549 cells infected with historic RSV strains (C) and contemporary RSV (D) strains. At least two independent experiments were performed, and ratios are presented as mean ± SD.

Finally, we probed the host response to RSV infection using a Luminex platform to measure levels of 29 cytokines and growth factors in the cell medium. Twenty-three different cytokines and growth factors were detected in response to RSV infection, all with levels showing strong differences between HEp-2 and A549 cells. Interferon type III (IL-29/IFN-λ) was not detected in RSV-infected HEp-2 cells but was found at high levels in RSV-infected A549 cells at 72 and 96 hpi. Interleukin (IL)-6 showed some of the highest increases relative to mock in both HEp-2 and A549 cells regardless of the infecting strain at 72 and 96 hpi (Fig. 12). IL-8 levels showed comparable increases at later time points in infected HEp-2 cells but, except for infections with RSV/A/Tracy, were greatly reduced in infected A549 cells (Fig. 12). Matrix metalloproteinase-9 (MMP-9), fibroblast growth factor-2 (FGF-2), granulocyte-macrophage colony-stimulating factor (GM-CSF), and tumor necrosis factor-α (TNF-α) showed much smaller increases than IL-6 and IL-8 but were the next highest detected in infected HEp-2 cells across RSV strains (Fig. 12A and B). Regulated upon activation, normal t cell expressed and presumably secreted (RANTES) showed increases comparable to or exceeding IL-6 in infected A549 cells (Fig. 12C and D). Infected A549 cells also showed significant increases in C-X-C motif chemokine ligand 11 (CXCL11) at 96 hpi from RSVA strains, and large increases in G-CSF and interferon gamma-induced protein 10 (IP-10) at 96 hpi from the more contemporary strains RSV/A/Ontario and RSV/B/Buenos Aires.

**FIG 13:**
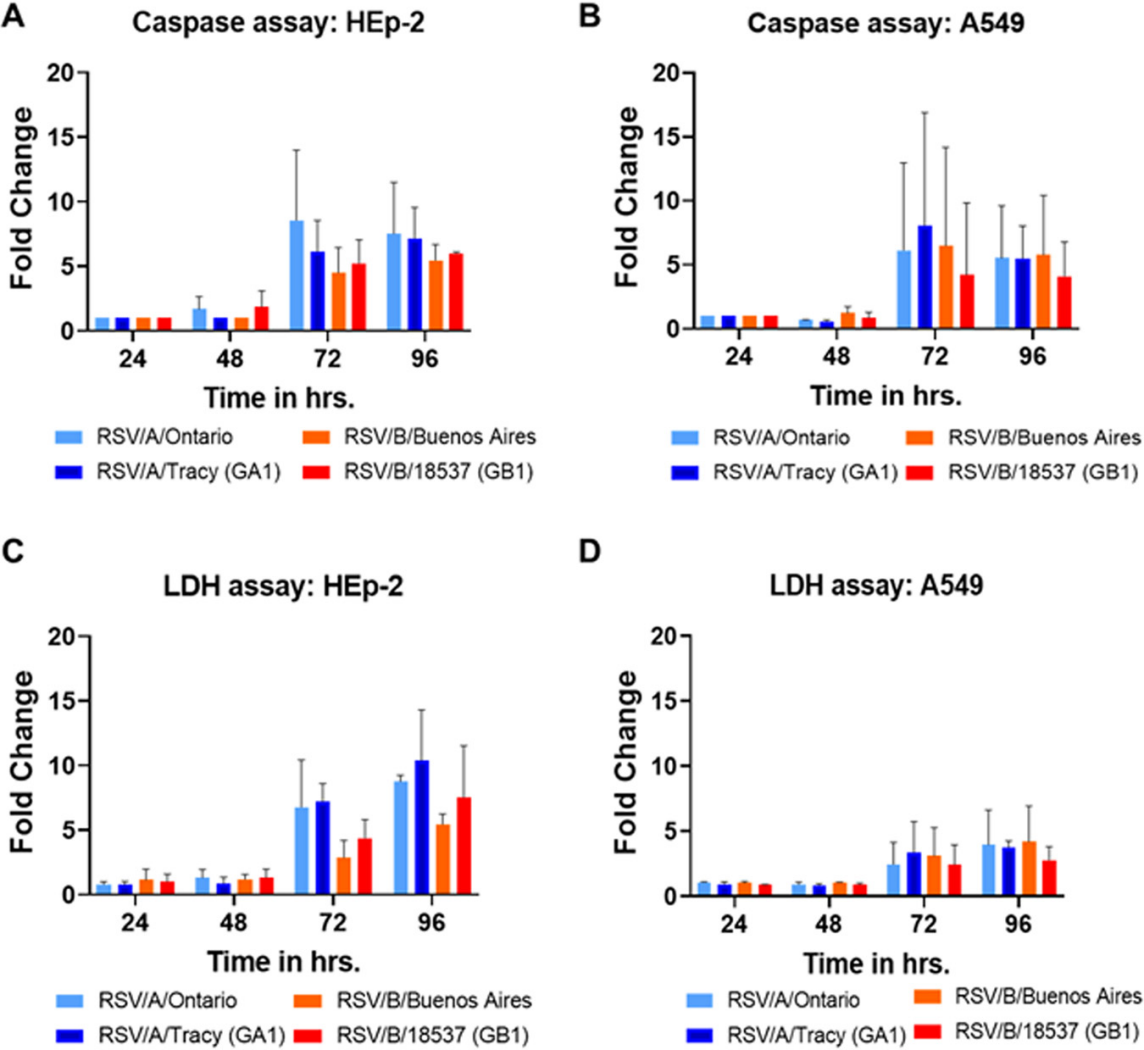
Caspase and lactate dehydrogenase (LDH) assays on HEp-2 and A549 cells infected with RSV strains. Cells were infected with RSV/A/Tracy (GA1), RSV/B/18537 (GB1), RSV/A/Ontario (ON), or RSV/B/Buenos Aires at a multiplicity of infection (MOI) of 0.01. The cultured supernatants were harvested from mock-infected and RSV-infected cells at 24, 48, 72, 96 h postinoculation (hpi). Fold changes of caspase released from (A) HEp-2 cells and (B) A549 cells are shown. Fold changes of LDH released from (C) HEp-2 cells and (D) A549 cells are shown. At least two independent experiments were performed, and ratios are presented as mean ± SD.

Similar to the cytokine kinetics observed in RSV-infected HEp-2 and A549 cells, the maximum fold-increase in lactate dehydrogenase (LDH) and caspase 3/7 levels relative to mock occurred at 72 and 96 hpi (Fig. 13). The relative fold increase in caspase 3/7 was comparable between infected HEp-2 and A549 cells (Fig. 13A and B) while the relative fold increase in LDH was approximately 2-fold higher in HEp-2 cells than A549 cells (Fig. 13C and D).

## DISCUSSION

Prior studies of RSV have focused mainly on RSV/A/A2 or RSV/A/Long, historic strains belonging to the same genotype (GA1) as the historic RSV/A strain used here (RSV/A/Tracy), in either HEp-2 or A549 cells (4–10, 16–18). The study presented here is the first to provide a direct comparison of the two major RSV subgroups and historic and contemporary strains in HEp-2 and A549 cells. We discovered a subtle, but potentially conserved, difference in viral gene expression between RSV subgroups A and B; and numerous differences in the host response to RSV infection between HEp-2 and A549 cell lines, including subtle but varied evidence of host cell line sensitivity to RSV genetic variation, especially between subgroups and between historic (no duplication in G gene) and contemporary (duplication in G gene) strains. In addition, there were major differences in cytokines/chemokines expression between infected HEp-2 and A549 cell lines with minor differences between historic and contemporary strains, and differences in viral infectivity and cellular damage profiles that were largely driven by cell type and to a lesser extent RSV strain. In total, these findings indicated that A549 cells can generate a robust antiviral response against historic and contemporary strains of RSV subgroups A and B compared to HEp-2 cells. The heterogeneous responses by the two cell types emphasize the potential for variation in different *in vivo* models of RSV infection, including humans. These diverse responses can help explain tissue- or organ-level variation observed within *in vivo* models of RSV infection. Finally, these findings are consistent with the host having a dominant role and the viral strain having a lesser role in the outcome of an RSV infection.

Measurements of viral replication kinetics revealed HEp-2 cells to be more permissive to RSV infection than A549 cells, giving rise to ∼10-fold more infectious virus at each of four time points collected over 96 hours. Perhaps consistent with their greater permissibility, confocal imaging revealed a greater cytopathic effect in RSV-infected HEp-2 than A549 cells at later time points (72 and 96 hpi). The two cell lines show global transcriptional responses to RSV infection that were similar in magnitude and kinetics but with a set of illuminating differences in the regulation of genes involved in several metabolic, immune, and cellular processes. In all, A549 cells gave rise to a more potently antiviral state than HEp-2 cells with much greater activation of genes upregulated by IFN-α and IFN-γ. The HEp-2 response to RSV infection was more proinflammatory. HEp-2 cells showed activation of genes involved in inflammasome production and earlier and more sustained activation of genes involved in the inflammatory response. In addition, HEp-2 cells produced elevated levels of IL-6, IL-8, and LDH in response to infection with any of the four RSV strains assayed, while A549 cells showed elevated levels of IL-6 and IL-8 only in response to RSV/A/Tracy.

Cytokine profiles for infected A549 cells showed elevated levels of interferon type III (IFN-lambda/IL-29); chemo-attractants like RANTES, IP-10, and MIP-1β; and IL-6. RSV-infected A549 cells also showed higher ratios of caspase 3/7 to LDH levels (relative to mock-infected cells) compared to RSV-infected HEp-2 cells. This is consistent with A549 cells manifesting a more potent antiviral (versus proinflammatory) response than HEp-2 cells to infection with RSV. These data translate to our observations in RSV-infected infants that a more robust antiviral state and a higher ratio of caspase to LDH are associated with less severe clinical outcomes (19, 20).

Levitz et al. (17) observed significantly increased levels of both IL-6 and RANTES in A549 cells at 24 hpi in response to an RSV/B strain lacking the G gene duplication (NH1125) versus an RSV/B strain with the G gene duplication (NH1067). We did not observe such a difference between RSV/B/18537 (lacking G gene duplication) and the RSV/B/Buenos Aires (containing G gene duplication) strains used here. However, the difference observed by Levitz et al. (17) decreases with decreasing MOI and became smallest at the lowest MOI (0.013) used. At 24 hpi, levels of both cytokines measured by both groups were quite low, being mostly well below a maximum of ∼50 picogram/mL. Our experiments were performed at a lower MOI (0.01), which was chosen to maximize the length of our experiment by delaying the onset of significant cytopathic effect.

Reverting to a general comparison of the two cell lines, it is interesting that A549 cells showed evidence of a transcriptional response favorable to the RSV life cycle at 48 hpi. For all infecting strains, genes involved in cholesterol biosynthesis (Fig. 8), fatty acid metabolism (Fig. 10), and adipogenesis (Fig. 10) were strongly activated. This upregulation might involve the production of additional host membranes in support of the generation of new virions. It might also support the production of additional cholesterol-rich rafts promoting further infection through enhanced virus binding to host (18) and release of new virus particles (21). Features of a host response that promote some aspects of the viral life cycle are not inconsistent with a broader host response that is more potently antiviral and ultimately reduces the permissibility of A549 cells to RSV infection.

The A549 cell line showed more instances of strain-dependence in its RSV response, suggesting heightened sensitivity to variation in infecting RSV strains. A549 cells also displayed an apoptotic response that rose monotonically to a peak at 96 hpi, while the HEp-2 apoptotic response was weaker and plateaued before dipping to a magnitude near baseline except in RSV/A/Ontario-infected HEp-2 cells, which showed strong repression of the apoptotic response at 96 hpi, suggesting a less coordinated and effective response from HEp-2 cells at resisting RSV infection (Fig. 8). Finally, it is worth remembering the origins of these cell lines. A549 cells were derived from a type II alveolar epithelial carcinoma while HEp-2 cells, which have been in use for decades (the line was originally derived over half a century ago from a larynx carcinoma), are widely known to have resulted from HeLa contamination (11–14). Thus, A549 cells would seem to be the more natural host of RSV infection. Moreover, the combination of an enhanced antiviral response plus some element of a response supporting RSV infection and the heightened sensitivity to RSV variation observed here suggest that RSV-infected A549 cells are more representative of natural RSV infection (i.e., RSV infection of whole human hosts).

A recent study similar to ours compared the host response to a single strain of RSV in A549 and BEAS-2B, a virus-transformed bronchial epithelial cell line, finding that BEAS-2B was less permissive to RSV infection and mounted a more potently antiviral response than the more proinflammatory response of A549 cells (4). These results are consistent with those reported here, but, because the authors used a single strain of RSV, BEAS-2B cell sensitivity to variation within RSV could not be assessed. If BEAS-2B cells are better representative of natural RSV infection (as A549 cells are versus HEp-2 cells), we would predict a comparable if not increased level of sensitivity to variation within RSV. We would also expect features of the BEAS-2B response to be in apparent support of the RSV life cycle because RSV has evolved to infect the respiratory epithelia.

Within each cell line, each of the four RSV strains, except for RSV/B/18537 at all time points but 24 hpi, replicated with comparable efficiency. However, cell lines did show some subtle sensitivity to infecting strain (mostly at subgroup and historic versus contemporary level) at the gross morphological level.

Unlike most of the strain-dependent differences reported here, the most striking difference observed in cell morphology occurred in HEp-2 cells. RSV/A-infected HEp-2 cells showed actin protrusions several microns in length extending from the cell surface at 96 hpi, and RSV/B-infected HEp-2 cells showed highly dense networks of actin filaments at the same time point. The origins of these morphological differences, both between RSV subgroups and the two cell lines, are unclear.

Transcriptional data and cytokine levels revealed a few instances of host sensitivity, mostly in A549 cells, to the infecting RSV strain. Genes upregulated in response to TGF-β1 showed a response that appeared strongly RSV subgroup-dependent in A549 cells (Fig. 9). In response to RSV/A infections, these genes were repressed until 72 hpi when they were strongly activated. In response to RSV/B infections, these genes remained repressed until 96 hpi when they were strongly activated. This difference seems to be largely due to differential regulation of the SMAD7 gene. Both the expression status of genes upregulated by STAT5 in response to IL-2 stimulation (Fig. 9) and genes involved in protein secretion (Fig. 10) showed an apparent dependence in A549 cells on whether the infecting RSV strain was historic (without G gene duplication) or contemporary (with G gene duplication). The latter also seems to hold in HEp2 cells. In both cell lines, genes associated with protein secretion were strongly repressed at 96 hpi only when the infecting strain was contemporary (Fig. 10). It is tempting to speculate that the duplication within the G gene of both strains (the G or attachment protein is expressed in both an integral membrane [IM] and a truncated non-IM form) leads to a similar host response with respect to the posttranslational processing of the G protein, potentially through the shared introduction of new glycosylation sites. Furthermore, production of the chemoattractant CXCL11 appeared subgroup-dependent in A549 cells, with RSV/A strains inducing higher levels of the chemokine than RSV/B strains. Mean levels of the proinflammatory cytokine IL-6, the maturational cytokine G-CSF, and the chemoattractant IP-10 were over 5-fold higher in response to contemporary versus historic RSV strains at 96 hpi in A549 cells, with much smaller differences if any at 72 hpi (except for IL-6 in response to RSV/A/Ontario) and earlier. These subtly different host responses highlight our lack of knowledge concerning the functional effects of well-known differences within RSV.

Measurements of viral gene expression revealed a single major difference in patterns of gene expression between subgroups A and B, with the two RSV/B strains supporting high transcriptional readthrough at the M-SH gene junction. This is predicted to result in lower expression of SH protein in cells infected with RSV/B strains due to increased production of dicistronic transcripts containing the SH ORF in a position distal to the 5′ cap (22, 23). This appears consistent with published phylogenetic trees suggesting weaker coevolution between RSV/B SH and the other RSV surface proteins (G and F) than RSV/A SH and G and F (24). Because the function of SH protein remains mysterious, it is not clear how this difference might relate to the few instances of a subgroup-dependent host response reported here. It is also important to note that readthrough at the M-SH gene junction can only directly contribute to the production of G transcripts through additional readthrough at the SH-G gene junction. From our data and others, the SH gene end signal is a very effective terminator of transcription (<5% readthrough). We estimate that only a small amount of polycistronic G-containing transcripts (∼3% of all G-containing transcripts) were produced via readthrough at the M-SH gene junction in RSV/B infections. Thus, high M-SH readthrough cannot account for observed G gene transcript levels that are much higher than those expected from sequential transcription with simple attenuation between genes.

Another apparent subgroup-dependent difference in transcriptional readthrough occurred at the NS1-NS2 gene junction. However, the standard deviations here for RSV/B are the highest measured for all four RSV strains and eight gene junctions. The source of this larger error was found in coverage plots showing a sudden drop in the number of reads mapping to the final third of the NS1 ORF in RSV/B infections. This conserved dip might result from the presence of a cryptic gene end (GE) signal. Regardless, its effect is to artificially increase both the average and standard deviation of the RSV/B NS1-NS2 readthrough percentages.

In summary, our broad and thorough characterization of *in vitro* RSV infections identified several viral and host-related features worth further study. These data revealed important differences in the host response to RSV infection between two widely used continuous cell lines and provided a baseline for *in vitro* studies using more realistic model systems of RSV infection, such as human airway organoids (10).

## MATERIALS AND METHODS

### Cell culture.

HEp-2 and A549 cells were cultured in minimum essential medium (MEM; Corning 10-010-CM), supplemented with 10% fetal bovine serum (HyClone SH30070.03), 1% of 10000 U/mL penicillin/streptomycin/25 μg/mL amphotericin B (Fungizone) (Gibco 15240062), 1% of l-glutamine (200 mM) (Gibco 25030081), and maintained in 5% CO_2_ at 36°C.

### RSV infection, qRT-PCR, and plaque assays.

Nearly confluent HEp-2 and A549 cells were infected with RSV/A/USA/BCM-Tracy/1989 (GA1), RSV/B/WashingtonDC.USA/18537/1962 (GB1), RSV/A/USA/BCM813013/2013 (ON), RSV/B/USA/BCM80171/2010 (BA) at an MOI of 0.01 for 1.5 h then the inoculum was removed, washed with PBS, and 2% FBS/MEM was added and incubated for a period of 24, 48, 72, or 96 hpi. Viral RNA was detected by qRT-PCR on cDNA generated with random hexamer primers and with qRT-PCR primers and probe targeting the nucleocapsid (N) gene of RSV, as previously described (25). Detected viral RNA includes mRNA, genomes, and antigenomes. Virus titers were measured by plaque assay as previously described (26).

### Statistical analysis of RSV replication kinetics data.

Separate linear regression models were made to test log-transformed (i) intracellular viral RNA levels, (ii) extracellular viral RNA levels, and (iii) levels of infectious virions (plaque forming units) for relationships with the following covariables: cell type, time, and RSV strain. A backward selection of covariables was conducted in each of the three analyses. Analyses were performed using R version 4.0.3.

### RNA sequencing analysis.

**(i) RNA isolation, library preparation, construction, and sequencing.**

RSV infections were performed on HEp-2 and A549 cell lines as described above and samples were collected at 24, 48, 72, and 96 hpi. For sample collection, host cells were lysed using TRIzol, and RNA was extracted using Mini Viral RNA kit (catalog no. 52904; Qiagen Sciences, Germantown, Maryland) and automated platform QIAcube (Qiagen, Hilden, Germany) according to the manufacturer’s instructions (25). All processing steps were performed by the Alkek Center for Metagenomics and Microbiome Research, Baylor College of Medicine, Houston, TX, USA. For mRNAseq, mRNA was enriched from total RNA using oligonucleotide (dT) beads. cDNA synthesis from both total RNA and mRNA-enriched RNA was performed using random hexamers and reverse transcriptase. The final cDNA library was created after purification, terminal repair, ligation of sequencing adapters, size selection, and PCR enrichment.

**(ii) RNA-sequencing analysis, quality control.** Fastq files were trimmed to remove adapters and base pairs that did not meet standards using Trim Galore (Version 0.4.1) (27, 28). Sequences were aligned against the human genome hg38 (GRCh38) using Hisat2 (version 2.1.0), sorted through samtools (version 1.5) (29, 30) and the count matrix was generated by mapping reads using featureCounts (version 1.6.0) (31). Nonoverlapping uniquely mapped features were counted for further analysis.

**(iii) Expression quantification and gene set enrichment analysis.** Differential gene expression (DGE) was determined using the R-packages edgeR and voom following published guidelines (16, 19). Count matrix was filtered for the coding genes, and a cutoff of 1 count per million (CPM) was used to remove low expression genes. The filtered counts were normalized using the trimmed mean of M-values (TMM). Genes were ranked according to their log_2_ gene expression for use in gene set enrichment analysis (GSEA version 3.0) and analysis was performed using ranked gene lists against the MSigDB database (version 6.1) (16, 19).

### Multiplex Luminex cytokine analysis.

Cytokine and chemokine levels from HEp-2 and A549 cell supernatants were determined using multiple Milliplex cytokine/chemokine magnetic bead panels (Millipore) according to the manufacturer’s instructions. Samples were assayed on the Magpix (Millipore) using xPonent software (Luminex). The kits used in this study include (i) Milliplex Human Cytokine Panel with Eotaxin/CCL11, FGF-2, G-CSF, GM-CSF, IL-1a, IL-1b, IL-6, IL-8/CXCL8, IL-17E/IL-25, IP-10/CXCL10, MCP-1, MCP-3, MIG, MIP1a, MIP1b, RANTES/CCL5, TNFa, VEGF-A, IL-33, TRAIL, TSLP, TAC/CXCL11, IL-29, BAFF, and HMGB1; (ii) TGFb1 Singleplex kit; (iii) Milliplex Human MMP Panel 2 with MMP9 and MMP7; and (iv) Milliplex Human TIMP Panel 2 with TIMP1.

### Lactate dehydrogenase (LDH) and caspase 3/7 analysis.

LDH and caspase 3/7 were measured in the supernatants of RSV-infected HEp-2 and A549 cells as previously described (20). In brief, total LDH activity was measured in the supernatant using the LDH Cytotoxic Detection Kit Plus, (Roche Applied Science, Indianapolis, IN, USA) following protocol instructions. l-lactate dehydrogenase (Roche Applied Science) was used to construct a standard curve that demonstrated linear dynamic range (*r* = 0.998) at the dilutions tested from 3.9 to 125 milliunits per milliliter (mU/mL). Caspase 3/7, a marker of apoptosis, was measured using the Caspase-Glo-3/7 kit (Promega, Madison, WI, USA). The luminescence assay was measured using a Biotek Synergy H1 microplate reader (Biotek) and reported as relative luminescence units (RLU). Purified caspase 3 from ENZO (catalog no. BML-SE169) was used as the standard for Caspase Glo 3/7 assays and Gen5 Imager Software was used for analysis.

### Immunofluorescence staining and high-resolution imaging.

Immunofluorescence staining was performed to identify changes in actin filament rearrangement after RSV infection in HEp-2 and A549 cells. HEp-2 and A549 cells were infected with RSV strains as described above on 96-well ibidi μ plates (ibidi, Germany catalog no. 89626). The plates were fixed with 4% paraformaldehyde and incubated at room temperature (RT) for 15 min at 24, 48, 72, or 96 hpi. The cells were permeabilized and blocked with 1% bovine serum albumin (BSA) with 0.1% Triton X-100 diluted in PBS for 30 min at RT. RSV was detected using an anti-RSV antibody from Abcam (catalog no. ab20745) at 1:1000 dilution in 1% BSA overnight incubation at 4°C. Nuclei and actin were stained with 4′, 6′-diamidino-2-phenylindole (DAPI) (1 μg/mL) and Alexa Fluor 568 Phalloidin (catalog no. A12380) at 1:1000 dilution, respectively, for 15 min at RT. High-resolution automated imaging was performed using a Cytiva DV Live epifluorescence image restoration microscope using an Olympus PlanApo N 60×/1.42 NA objective and a 1.9kx1.9k pco.EDGEsCMOS 5.5 camera with a 1024 × 1024 FOV. The filter sets used were DAPI (390/18 excitation, 435/48 emission), TRITC (542/27 excitation, 594/45 emission), and Cy5 (632/22 excitation, 676/34 emission). Z stacks (0.2 μm) covering the whole cell (∼10 μm) were acquired before applying a conservative restorative algorithm for quantitative image deconvolution using SoftWorx v7.0 and saving images as max pixel intensity projections.

### Data availability.

The RNA-Seq and mRNA-Seq data are available in GEO under accession number GSE196385.
